# Three-Dimensional Point Cloud Reconstruction of Unstructured Terrain for Autonomous Robots

**DOI:** 10.3390/s25164890

**Published:** 2025-08-08

**Authors:** Wei Chen, Xiufang Lin, Xiangpan Zheng

**Affiliations:** 1 College of Physics and Electronic Information Engineering, Minjiang University, Fuzhou 350108, China; 2 Pen-Tung Sah Institute of Micro-Nano Science and Technology, Xiamen University, Xiamen 361005, China

**Keywords:** 3D reconstruction, unstructured terrain, autonomous robots, LiDAR

## Abstract

In scenarios such as field exploration, disaster relief, and agricultural automation, LIDAR-based reconstructed terrain models can largely contribute to robot activities such as passable area identification and path planning optimization. However, unstructured terrain environments are typically absent and poorly characterized by artificially labeled features, which makes it difficult to find reliable feature correspondences in the point cloud between two consecutive LiDAR scans. Meanwhile, the persistence of noise accompanying unstructured terrain environments also causes certain difficulties in finding reliable feature correspondences between two consecutively scanned point clouds, which in turn leads to lower matching accuracy and larger offsets between neighboring frames. Therefore, this paper proposes an unstructured terrain construction algorithm combined with graph optimization theory based on LOAM algorithm further introducing the robots motion information provided by IMU. Experimental results show that the method proposed in this paper can achieve accurate and effective reconstruction in unstructured terrain environments.

## 1. Introduction

With the rapid development of unmanned robots, geohazard monitoring, and field exploration, the demand for high-precision 3D reconstruction of complex unstructured terrain is becoming increasingly urgent [1]. Unstructured terrain, such as mountains, ruins, forests, etc., is characterized by irregular surface morphology, severe occlusion, and sparse texture features [2]. Traditional 3D reconstruction methods based on GPS or preset markers have significant limitations in complex environments, resulting in sparse reconstructed point clouds, much noise interference, and low efficiency [3]. How to achieve efficient and robust 3D point cloud reconstruction of unstructured terrain has become a research hotspot in the fields of computer vision, autonomous robot navigation, and environment perception [4].

Three-dimensional reconstruction can be mainly divided into two research directions, LiDAR map construction [5] and visual map construction [6], depending on the different types of sensors. Visual sensor-based map construction has been widely utilized due to its low energy consumption and light weight [7]. Boston Dynamics employs a combination of binocular vision and sensors such as IMUs to achieve autonomous exploration of unknown environments in the wild by robot dogs. Cheng Y et al. investigated visual sensor-based environment modeling to provide critical Mars surface terrain information for motion control as well as path planning of the rover [8]. However, the accuracy of visual sensor modeling is relatively low, in contrast to LiDAR sensor-based map construction that can reach millimeter-level accuracy and is more widely adopted in applied research on environmental reconstruction [9].

The crucial factor in LIDAR-based environment modeling is the process of data association and matching between neighboring point cloud frames [10]. A typical method to identify the corresponding transformation relationship between two neighboring frames of LiDAR scanned point cloud is the ICP algorithm [11]. The algorithm iteratively aligns the two point clouds by finding the correspondence of each point between the two frames until a set threshold is reached and then outputs the transformation parameters between the two point clouds. However, when the scanned point cloud is of a large order of magnitude, the algorithm employed results in excessive computational cost and poor timeliness [12].

**Figure 1:**
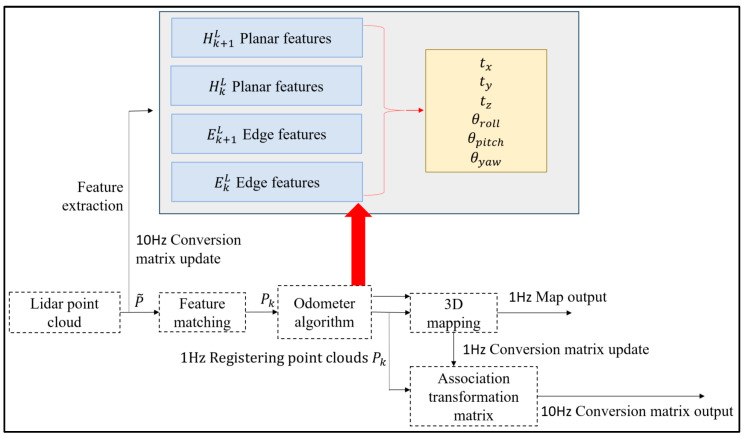
LOAM mapping framework with laser odometer.

Zhang et al. proposed the LOAM real-time laser odometry and construction algorithm [13], whose algorithm focuses on matching the estimated state information in order to further realize the real-time construction of maps. When employing LiDAR sensing sensors for mapping research and motion estimation, the LOAM algorithm offers several benefits, including high accuracy and strong real-time performance. The map construction problem is split into two distinct processes by this algorithm, which runs concurrently. A range algorithm is implemented at a higher frequency by an algorithm to estimate the single line LiDAR’s motion state. As illustrated in Figure 1, an alternative technique matches point clouds at lower frequencies with accuracy to produce real-time environmental mapping.

In recent years, with the development of multi-sensor fusion applications and deep learning, LOAM algorithms have evolved a variety of optimization algorithms. LeGO-LOAM splits a single-frame point cloud into subframes for parallel matching and shortens the processing delay [14]. LO-Net convolutional network jointly learns inter-frame matching with position and attitude estimation, and implicitly handles dynamic objects through mask-weighted geometrically constrained loss with accuracy comparable to LOAM and better generalization performance [15]. CSS-LOAM replaces traditional curvature computation with curvature scale space algorithms to analyze multi-scale geometric properties of point clouds with 40% computational efficiency improvement [16]. KDD-LOAM constructs keyframe KD trees for millisecond feature retrieval in loopback detection [17]. However, the majority of such algorithms show better performance and accuracy in structured environments, which are distorted to a certain extent in the face of unclear environmental features of unstructured terrain and large fluctuations in robot positional attitude caused by terrain excitation. Therefore, in this paper, we take the algorithm LOAM as the basis to explore the accurate environment modeling method for unstructured terrain.

However, in order to gather more precise and thorough three-dimensional environmental information, the LOAM algorithm often adopts a stop-scan-forward motion using a single line LiDAR. This results in subpar real-time performance across the system. Additionally, robots operating in complicated unstructured terrain conditions frequently experience unstable motion because of the nonlinear stimulation of the ground on the tires [18]. This will result in the distortion of LiDAR-scanned point cloud data, which will have a significant impact on the subsequent data correlation. Finding trustworthy feature correspondences in the point cloud between two consecutive LiDAR scans is further complicated by the absence of manually labeled features and ambiguous features in unstructured terrain environments [19]. This paper presents IMU motion information in response to the aforementioned features, with the aim of mitigating the distortion impact of scanned point clouds. In addition, we present graph optimization theory to enhance and optimize the conventional LOAM algorithm, accomplishing an accurate reconstruction of unstructured terrain.

**Figure 2:**
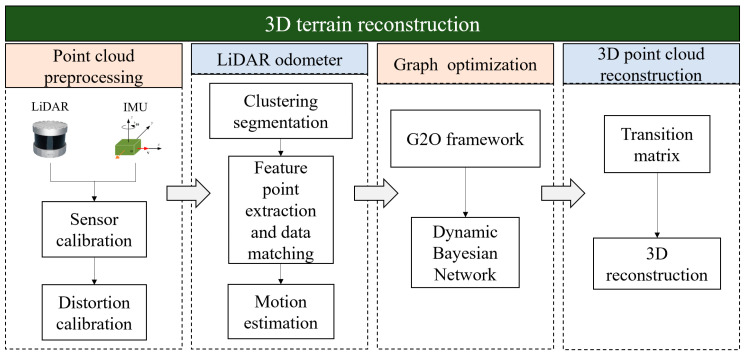
LiDAR based 3D unstructured terrain reconstruction framework.

Figure 2 displays the foundation for 3D terrain reconstruction using LiDAR data. First, calibrate the LiDAR and IMU. Then, use the motion information from the IMU to lessen the distortion effect of the scanned point cloud. To perform LiDAR motion estimation, next processes include clustering and segmenting the scanned point cloud, extracting feature points, and finishing data matching. In order to optimize the mapping technique and accomplish the reconstruction of unstructured terrain environments, the graph optimization theory is finally introduced under the G2O framework.

## 2. Preprocessing of Point Clouds Scanned by LiDAR

LiDAR is relatively insensitive to light compared to visual sensors that can be used stably even at night when there is insufficient light [20]. In addition, the high resolution of LiDAR permits it to capture details of the environment at remote distances over a wide field of view. The error of LiDAR is relatively constant whether the distance measured is remote or not [21]. Therefore, robot-mounted LiDAR is used as an environment-aware sensor for reconstruction in this paper. However, the LiDAR is in motion along with the movement of the robots. The movement-induced distortion of the ambient 3D point cloud data may usually be disregarded if the LiDAR scan speed is significantly faster than the speed of the moving robots [22]. In this case, the standard ICP algorithm can be utilized for matching between different scanned frames to achieve better matching performance. Meanwhile, compensation following velocity estimation based on ICP can reduce LiDAR motion-induced distortion. However, if the scanning motion of LiDAR is relatively slow, especially in unstructured terrain environment where the robots are often accompanied by unsteady motion, it will further result in the distortion of the scanned point cloud affecting the accuracy of the algorithms. Therefore, the pre-processing of the LiDAR scanned point cloud is required. In this case, this paper introduces the IMU motion information for aberration calibration of the acquired raw point clouds.

### 2.1. Calibration of LiDAR Distortion

One potential source of distortion in a single-frame point cloud obtained through LiDAR scanning is the robot-mounted LiDAR, which tracks the motion of the robots. Unpredictable and variable terrain, especially in unstructured terrain conditions, results in sudden shifts in robots’ motion, which worsens the distortion of the point cloud data.

Independent position estimation is a commonly used approach to address LiDAR distortion. The odometer is another kind of measurement technique. For instance, we calibrate it by using an encoder mounted on the wheel for motion estimation. Wheel encoders are inappropriate in unstructured terrain due to the propensity of wheels to slip. Therefore, in this paper, the robots motion data provided by the IMU is introduced in the initial stage of the point cloud processing to eliminate the point cloud distortion caused by nonlinear motion.

**Figure 3:**
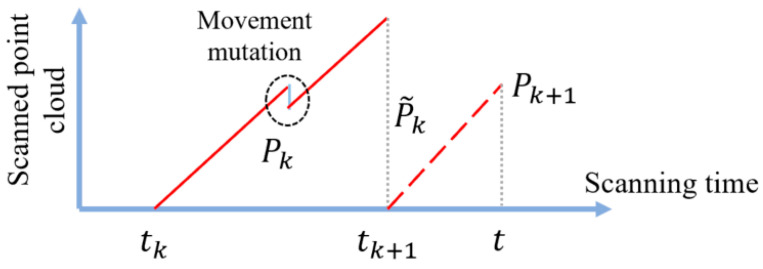
Two consecutive frames of point cloud scanned by LiDAR.

Motion compensation is another way of explaining the elimination of distortion. Concurrently map every point in the constantly scanned point cloud $P_{k}$ to the same time $t_{k+1}$. As illustrated in Figure 3, calibrate the posture increment during this scanning phase during [$t_{k+1}$, t ] between the gradually detected point cloud $P_{k+1}$ and the final scanned point cloud $P_{k}$.

**Figure 4:**
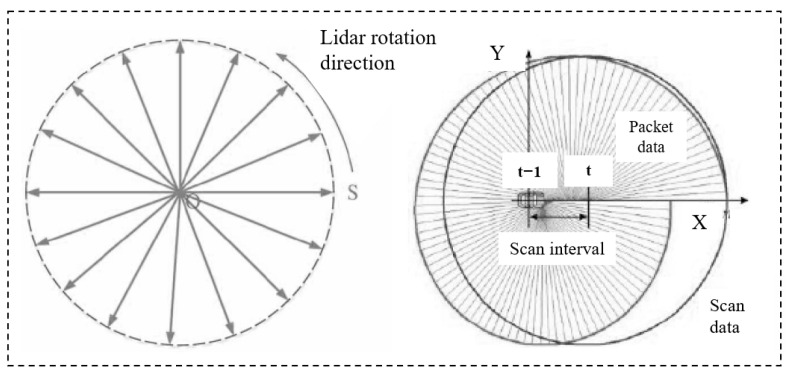
LiDAR data calibration following robots’ movement.

The data acquisition frequency of the LiDAR is set to 10 Hz to maintain the data density of the acquisition and the effective operation of real-time data processing. A single frame of LiDAR data acquisition is separated by 100 milliseconds between the beginning and the end of the frame. In the same point cloud frame, the attitude of the body will be changed. Therefore, as the LiDAR is moved with the robots, each scan line will no longer be a traditional circle, but a spiral line as shown in Figure 4. Segmentation may occur to varying degrees when there is a sudden change in robots’ motion, and therefore it is necessary to compensate for this in conjunction with the attitude information of the robots’ motion.

Compared with 10 Hz LiDAR data, the IMU’s acquisition frequency is 100 Hz, which can effectively eliminate the data distortion caused by LiDAR’s movement with the robots, and better compensate for the loss of LiDAR information during the scanning process. It is important to note that all IMU information throughout the scanning period is collected during the point cloud preprocessing phase of the point cloud data motion distortion calibration procedure.

**Figure 5:**
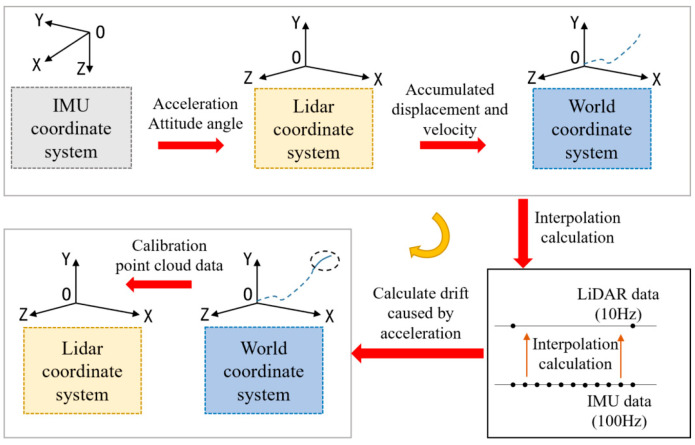
Calibrating Lidar point cloud distortion procedure.

Figure 5 illustrates the method of calibrating LiDAR point cloud distortion. Initially, the acceleration and Euler angles measured by the IMU were converted to the LiDAR coordinate system using the transformation relations identified during the calibration process. Then, the acceleration from the LiDAR coordinate system is converted to the world coordinate system utilizing the Euler Angle Transform, and the acceleration data from the IMU is utilized to calculate the cumulative displacement and velocity.

(1) $$S_{k+1}=S_{k}+\frac{1}{2}\times a\times ∆t^{2}$$

(2) $$V_{k+1}=V_{k}+a\times ∆t$$

The main contributor to the distortion is the drift caused by the unstable motion of the LiDAR. Attitude measurements during LiDAR accelerated motion are calculated by interpolating the IMU data in this paper.

The sampling frequencies of the LiDAR and IMU are 10 Hz and 100 Hz, respectively. This determines the timestamps of the two IMU data points corresponding to the start and end of the scan, as well as the timestamp of the current LiDAR scan frame. The displacement produced by the accelerated motion from the current frame of the LiDAR point cloud to the current scan time is:

(3) $$S_{p}=S\;-\;V\;\times \nabla T$$

In the formula above, the drift displacement is denoted by $S_{p}$, and the total displacement is denoted by S.

The calculated values are referenced to the world coordinate system and require an additional conversion to the LiDAR coordinate system in order to compensate for the corresponding LiDAR point cloud data to achieve an aberration calibration of the point cloud.

### 2.2. Three-Dimensional Point Cloud Feature Extraction

Since unstructured terrain environments contain common and relatively smooth terrain, planar segmentation of the original point cloud is performed to speed up the computational efficiency of the algorithm. The original point cloud is initially downsampled by the VoxelGrid to reduce the amount of data and maintain the overall geometric and topological properties of the point cloud. The normal is then utilized as a local descriptor of the point cloud to seed a number of points with minimal curvature for growth clustering in order to cluster points that satisfy a curvature smoothing threshold to form planar blocks. Finally, the plane is fitted utilizing a random sampling consistent iterative algorithm.

For the dense point cloud generated, it is essential to ensure that enough stable feature points can be efficiently extracted to fulfill the alignment requirements. The parameter settings of the feature point detection algorithm are shown in Table 1, and all the parameters are determined and approved through a large number of experiments to guarantee the high efficiency and stability of the algorithm.

The point cloud is further segmented in order to improve processing efficiency and feature extraction accuracy. Suppose $P_{t}\;=\;\{p_{1},\;\;p_{2},\;\ldots ,\;p_{n}\}$ is the original single frame point cloud acquired at time t. $p_{i}$ is a point in the single-frame point cloud $P_{t}$. $P_{t}$ is initially projected onto the distance image, and $r_{i}$ associated with $p_{i}$ represents the Euclidean distance from the corresponding point $p_{i}$. Then, the points are clustered utilizing a distance-based clustering segmentation method and independent labels are assigned to the points in the same class. The original point cloud contains numerous independent noisy points at the same time, which may produce unreliable features, in order to perform fast and reliable feature extraction in this paper clustering with less than a specific number of thresholds is ignored. Once planar segmentation has been performed, only points that may represent large objects are retained for further subsequent processing.

To create a single frame point cloud $P_{k}$, LiDAR scans the surrounding at a frequency of 10 Hz. Edge and planar points are chosen to be feature points in the laser point cloud $P_{k}$. Let $p_{i}$ represent a point in $P_{k}$, $p_{i}\in P_{k}$. Considering Q is the collection of continuous points $p_{i}$ that the same scanning line’s LiDAR collected. Q will comprise half of the points on either side of $p_{i}$ when a complete point cloud is obtained, with an angle separation of 0.2° between neighboring points. Define an expression for evaluating the smoothness S of a localized surface as follows:

(4) $$S=\frac{1}{\left|Q\right|\left\|X_{\left(k,i\right)}^{L}\right\|}\left\|\sum_{j\in Q\;i\neq j}\left(X_{\left(k,i\right)}^{L}-X_{\left(k,j\right)}^{L}\right)\right\|.$$

The coordinates of the i-th point $p_{i}$ in the point cloud $P_{k}$ in the LiDAR origin coordinate system are represented by $X_{\left(k,i\right)}^{L}$ in the formula above.

**Figure 6:**
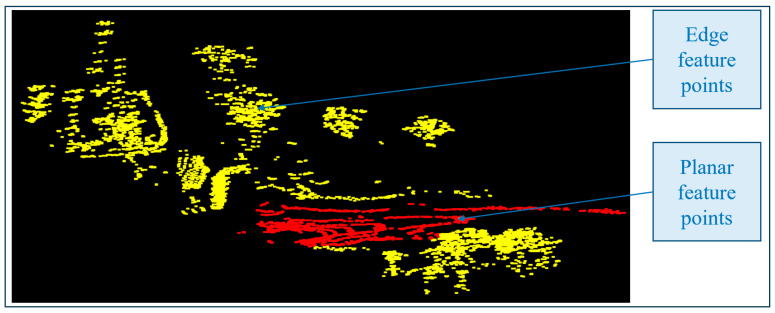
Planar and edge feature points were taken from a single frame point cloud.

Sort the points in the same scan line based on the smoothness value of the local surface and select feature points. The threshold $S_{th}$ is used to distinguish different types of feature points. Points with smaller S values are chosen as planar feature points, whereas ones with larger S values are chosen as edge feature points. Figure 6 displays the feature point extraction results from the point cloud. The red and yellow spots correspond to the planar feature points and edge feature points, respectively.

### 2.3. Matching of Point Cloud Feature Points

The parameter settings of the feature point alignment algorithm are shown in Table 2, and all the parameters are determined through a large number of experiments to ensure the high efficiency and stability of the algorithm.

Meanwhile, assuming $t_{k}$ is the start scanning time of the current point cloud frame $P_{k}$. LiDAR scanned a single frame point cloud, which is represented as $P_{k}$. Additionally, ${\tilde{P}}_{k}$ represents the point cloud at time $t_{k+1}$ where it is reprojected. Combine the recently obtained point cloud $P_{k+1}$ and ${\tilde{P}}_{k}$ inside the next scanning period $t_{k+1}$ to estimate the LiDAR’s motion status. Moreover, the set of planar feature points as $H_{k+1}$ and the set of edge feature points as $E_{k+1}$ from the $P_{k+1}$ using the previously mentioned technique. Determine which edge lines in ${\tilde{P}}_{k}$ match the points in $E_{k+1}$, and which planar blocks in ${\tilde{P}}_{k}$ match the points in $H_{k+1}$.

Assuming that $T_{k+1}^{L}$ is the six degrees of freedom position and that attitude transformation corresponds to the LiDAR in time $\left[t,t_{k+1}\right]$:

(5) $$T_{k+1}^{L}={\left[t_{x},t_{y},t_{z},\theta_{x},\theta_{y},\theta_{z}\right]}^{T}$$

where the translation distance along the *X*, *Y*, and *Z* axes is represented by the values $t_{x}$, $t_{y}$, and $t_{z}$. The *X*, *Y*, and *Z* axes’ rotation angle information for $\theta_{x}$, $\theta_{y}$, and $\theta_{z}$.

Being calibrated using the IMU, LiDAR motion can be roughly assumed to remain constant for a certain amount of time. The calibration to be performed above is as expressed in Section 2.1. In this paper, the LiDAR is initially calibrated with the IMU. Then, we refer to the six degrees of freedom information of vehicle motion provided by the IMU and combine it with the LiDAR point cloud to eliminate the point cloud distortion due to nonlinear motion. Prior to the point cloud data calibration process, all six degrees of freedom information of the IMU for that scan time was already available. Finally, the accumulated displacements and velocities are calculated according to the information of the six degrees of freedom of the IMU to form a complete point cloud which is closer to the real unstructured environment.

The position and attitude transformation between $T_{\left(k+1,i\right)}^{L}$ and [$t_{k+1}$, $t_{i}$], for a given point $\left(p_{i}\in P_{k+1}\right)$ and time $t_{i}$, can then be used to determine $T_{\left(k+1,i\right)}^{L}$ using linear interpolation of $T_{k+1}^{L}$:

(6) $$T_{\left(k+1,i\right)}^{L}=\frac{t_{i}-t_{k+1}}{t-t_{k+1}}T_{k+1}^{L}$$

The collection of edge points and plane points that $P_{k+1}$ extracted, and the set of points that $E_{k+1}$ and $H_{k+1}$ reprojected at time $t_{k+1}$, are denoted by ${\tilde{E}}_{k+1}$ and ${\tilde{H}}_{k+1}$, respectively. Between $E_{k+1}$ and ${\tilde{E}}_{k+1}$ or $H_{k+1}$ and ${\tilde{H}}_{k+1}$, the conversion connection is:

(7) $$E_{\left(k+1,i\right)}^{L}=R{\tilde{E}}_{\left(k+1,i\right)}^{L}+T_{\left(k+1,i\right)}^{L}\left(1:3\right),$$

(8) $$H_{\left(k+1,i\right)}^{L}=R{\tilde{H}}_{\left(k+1,i\right)}^{L}+T_{\left(k+1,i\right)}^{L}\left(1:3\right).$$

The coordinates of $E_{k+1}$ and $H_{k+1}$ midpoint $p_{i}$ in the LiDAR origin coordinate system are represented by $E_{\left(k+1,i\right)}^{L}$ and $H_{\left(k+1,i\right)}^{L}$. *R* is the rotation matrix, and $T_{\left(k+1,i\right)}^{L}(1:3)$ stands for the first to third rows of $T_{\left(k+1,i\right)}^{L}$:

(9) $$R=R_{z}R_{x}R_{y}=\left[\begin{array}{c} \cos\theta_{z}\;\;\;\;\;\;-\sin\theta_{z}\;\;\;\;\;\;\;0\\ \sin\theta_{z}\;\;\;\;\;\;\;\;\;\;\;\cos\theta_{z}\;\;\;\;\;\;\;0\\ \;\;\;0\;\;\;\;\;\;\;\;\;\;\;\;\;\;\;\;\;\;\;\;0\;\;\;\;\;\;\;\;\;\;\;\;1 \end{array}\right]\left[\begin{array}{c} 1\;\;\;\;\;\;\;\;\;\;\;0\;\;\;\;\;\;\;\;\;\;\;\;\;\;\;\;0\;\;\;\;\;\\ 0\;\;\;\;\;\;\cos\theta_{x}\;\;\;\;\;-\sin\theta_{x}\\ 0\;\;\;\;\;\;\sin\theta_{x}\;\;\;\;\;\;\;\;\;\cos\theta_{x} \end{array}\right]\left[\begin{array}{c} \;\;\;\;\;\cos\theta_{y}\;\;\;\;\;0\;\;\;\;\;\;\;\sin\theta_{y}\\ \;\;\;\;\;\;\;\;\;0\;\;\;\;\;\;\;\;\;1\;\;\;\;\;\;\;\;\;\;\;\;0\;\;\;\\ \;\;-\sin\theta_{y}\;\;\;\;\;0\;\;\;\;\;\;\;\cos\theta_{y} \end{array}\right]$$

The rotation angle is denoted by θ in the equation above,

(10) $$\theta =\|T_{\left(k+1,i\right)}^{L}\left(4:6\right)\|$$

It can be concluded that:

(11) $${\tilde{E}}_{\left(k+1,i\right)}^{L}=R^{-1}\left(E_{\left(k+1,i\right)}^{L}-T_{\left(k+1,i\right)}^{L}\left(1:3\right)\right),$$

(12) $${\tilde{H}}_{\left(k+1,i\right)}^{L}=R^{-1}\left(H_{\left(k+1,i\right)}^{L}-T_{\left(k+1,i\right)}^{L}\left(1:3\right)\right).$$

Next, for every point in ${\tilde{E}}_{k+1}$ and ${\tilde{H}}_{k+1}$, find the closest neighboring point in ${\tilde{P}}_{k}$. If $p_{j}$ and $p_{l}$ are the respective edge lines for the edge point $p_{i}\in {\tilde{E}}_{k+1}$ in the point cloud ${\tilde{P}}_{k}$, then the equivalent distance is:

(13) $$d_{E}=\frac{\left|\left({\tilde{E}}_{\left(k+1,i\right)}^{L}-{\tilde{E}}_{\left(k,j\right)}^{L})\times ({\tilde{E}}_{\left(k+1,i\right)}^{L}-{\tilde{E}}_{\left(k,l\right)}^{L}\right)\right|}{\left|{\tilde{E}}_{\left(k,j\right)}^{L}-{\tilde{E}}_{\left(k,l\right)}^{L}\right|}.$$

The coordinates of points $p_{i}$, $p_{j}$, and $p_{j}$ are denoted by the symbols ${\tilde{E}}_{\left(k+1,i\right)}^{L}$, ${\tilde{E}}_{\left(k,j\right)}^{L}$, and ${\tilde{E}}_{\left(k,l\right)}^{L}$ in the formula above.

For the planar point $p_{i}\in {\tilde{H}}_{k+1}$, if $p_{j}$, $p_{l}$, and $p_{m}$ are the corresponding planar blocks in the point cloud ${\tilde{P}}_{k}$, the corresponding distances are:

(14) $$d_{H}=\frac{\left|\left({\tilde{H}}_{\left(k+1,i\right)}^{L}-{\tilde{H}}_{\left(k,j\right)}^{L})[({\tilde{H}}_{\left(k,j\right)}^{L}-{\tilde{H}}_{\left(k,l\right)}^{L})\times ({\tilde{H}}_{\left(k,i\right)}^{L}-{\tilde{H}}_{\left(k,m\right)}^{L})\right]\right|}{\left|\left({\tilde{H}}_{\left(k,j\right)}^{L}-{\tilde{H}}_{\left(k,l\right)}^{L})\times ({\tilde{H}}_{\left(k,j\right)}^{L}-{\tilde{H}}_{\left(k,m\right)}^{L}\right)\right|}.$$

The coordinates of $p_{i}$, $p_{j}$, $p_{l}$, and $p_{m}$ are represented by the letters ${\tilde{H}}_{\left(k+1,i\right)}^{L}$, ${\tilde{H}}_{\left(k,j\right)}^{L}$, ${\tilde{H}}_{\left(k,l\right)}^{L}$, and ${\tilde{H}}_{\left(k,m\right)}^{L}$ in the formula above.

## 3. Algorithm for 3D Reconstruction

### 3.1. Point Cloud Feature Matching for 3D Reconstruction

**Figure 7:**
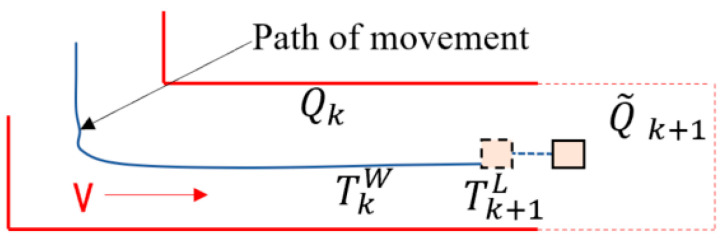
An illustration of the 3D reconstruction schema.

Figure 7 displays the schematic diagram for 3D reconstruction. The LiDAR posture $T_{k}^{W}$ produced by the algorithm at time k is represented by the blue curve. The LiDAR motion data $T_{k+1}^{L}$ computed with the previously described laser mileage calculation method is represented by the blue dotted line. Project the calibrated point cloud onto the red area of $Q_{k}$ that is depicted on the current map after obtaining $T_{k}^{W}$ and $T_{k+1}^{L}$. On the map, the calibrated point cloud is shown as ${\tilde{Q}}_{k+1}$.

The detailed procedure is as follows:

Firstly, convert the global coordinate system from the calibrated point cloud, and then match features with the local map that was created. Point clouds outside of the range will be eliminated upon surpassing the predetermined threshold range. Secondly, based on the smoothness *S* of the nearby surface, ascertain if the point is a plane feature point or an edge feature point. Finally, locate the feature points’ matching points on the created local map $Q_{k}$. Determine the plane block of the matching plane formed by the three nearest neighbor plane points, or the edge line of the corresponding edge formed by the two nearest neighbor edge points. Accurately match newly contributed point clouds to the locally created map. Simultaneously, the attitude determined by the laser mileage computation method was slightly modified.

When compared to the laser mileage calculation approach, the reconstruction algorithm in this paper operates at a very low frequency. The laser odometer operates at a frequency of 10 Hz, whereas the reconstruction process operates at 1 Hz. When ${t=t}_{k+1}$, LiDAR scanning produces an undistorted point cloud frame ${\tilde{P}}_{k+1}$ and concurrently produces motion pose change information $T_{k+1}^{L}$ from the LiDAR. At the end of the scanning time $t_{k}$, the attitude of the LiDAR on the local map is denoted by $T_{k}^{W}$, and the generated map point cloud gathered to that point is specified as $Q_{k}$. The point cloud of ${\tilde{P}}_{k+1}$ represented by ${\tilde{Q}}_{k+1}$, and it is projected onto the global coordinate system. Next, use the LiDAR’s attitude $T_{k+1}^{W}$ to optimize the match between $Q_{k}$ and ${\tilde{Q}}_{k+1}$.

Determine the matching relationship between feature points first. To facilitate point retrieval, store the point cloud $Q_{k}$ in a designated cubic region with a predetermined edge length. Then, extract the points that correspond to the ${\tilde{Q}}_{k+1}$ feature point and store them in an octree. The covariance matrix of O is A, assuming that O is a collection of surrounding points inside the area surrounding the feature points identified in $Q_{k}$. A has an eigenvalue of and an eigenvector of $\vec{x}$. λ has two larger and one smaller eigenvalue if O is on a plane block. The minimal eigenvalue of a feature vector corresponds to the direction of a planar block. λ has one larger and two smaller eigenvalues if O is on the edge. The feature vector that corresponds to the largest eigenvalue represents the edge line’s orientation. Three points are chosen on the plane block, or two points are chosen on the edge line and fitted using the L-M method, then we add ${\tilde{Q}}_{k+1}$ to the locally created map. This process is performed in order to further calculate the distance between feature points and their corresponding points.

### 3.2. An Algorithm for Modeling Optimization Derived from Graph Optimization Theory

**Figure 8:**
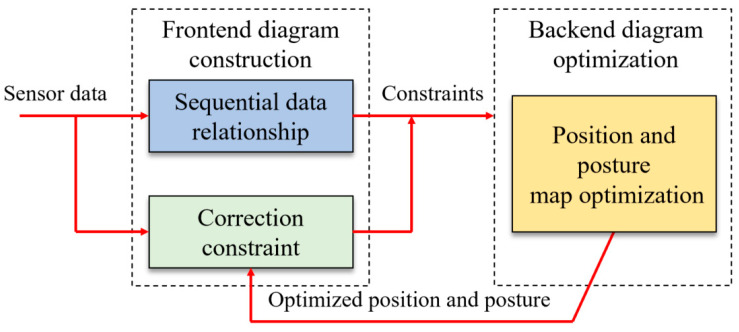
Diagrammatic representation of the optimization process.

The accuracy of reconstruction cannot be guaranteed when using data association matching between two point clouds, as this might easily result in inaccurate matching and error accumulation [23]. Graph optimization can solve drift and erroneous matching while reducing cumulative errors even further, increasing the algorithm’s adaptability [24]. In order to achieve complete motion trajectory estimation of robots while preserving global consistency in map construction, this algorithm determines the relationship between the historical pose state of the robots and the current observation information, and forms correction constraints between the current pose and the historical pose. A backend graph optimization processing approach is presented in this article with the ultimate goal of improving local map creation outcomes. Figure 8 shows the process of graph optimization.

It can be broken down into the subsequent steps:

#### 3.2.1. Converting the Real-Time Reconstruction Issue into a Description-Based Least Squares Problem

The fundamental task of real-time reconstruction is to solve the maximum posterior probability estimation of the position of feature points and the pose of moving robots on the map based on the observer’s output and the motion state data analyzed by the LiDAR mileage calculation method, as indicated by the following equation:

(15) $$X^{*}=argmaxP\left(X|U\right).$$

X in the formula above stands for the estimated pose information of moving robots and the position information of feature points on the map. The motion control input $u_{i}$ and the corrective constraint result $u_{ij}$ are included in the parameter *U*. To solve the joint probability distribution on the right side of the equation above, break it down as follows:

(16) $$P(X|U)\propto \prod_{i}P\left(x_{i+1}|x_{i},u_{i}\right)\prod_{ij}P\left(x_{j}|x_{i},u_{ij}\right).$$

Determine the logarithm of the two sides of the preceding equation by breaking down the posterior probability estimation into closed-loop constraints and motion information model constraints:

(17) $$X^{*}=argmaxP\left(X|U\right)=argmin\left\{\sum_{i}{\left\|f\left(x_{i},u_{i}\right)-x_{i+1}\right\|}_{\sum_{i}\;}^{2}+\sum_{ij}{\left\|f\left(x_{i},u_{ij}\right)-x_{j}\right\|}_{A_{ij}}^{2}\right\}.$$

At this stage, the least squares optimization problem will be solved in place of the real-time reconstruction.

#### 3.2.2. Fundamental Structure Derived from Graph Optimization Theory

Real-time reconstruction is modeled using graph models algorithm based on graph optimization theory. It utilizes observed data to maximize the estimation of a moving robot’s whole motion trajectory and produces mapping results that are globally consistent. In the graph model, the edges stand in for constraint connections, and each node represents the system state of moving robots and the environment at various moments in time.

Graph optimization theory separates the real-time reconstruction problem into two parts: the back-end, which further optimizes the graph model created by the front-end, and the front-end, which creates graph models based on observation and system constraints. The front-end data association pertains to the assessment of the relative attitude of robots and the inter-frame matching of neighboring data. From the standpoint of data processing, the main goal of this process is to address the problem of data correlation. Graph optimization takes into account global data correlation without directly processing observed data. From the optimization process’s point of view, it is dependent on the observed data to determine the ideal relationship between local graphs and finish the construction of the graph.

The front-end’s graph will not match the global graph because of mistakes and noise in observations. In an ideal state, all of the following criteria must be satisfied for there to be a fully closed loop:

(18) $$T_{0}T_{1}\cdot \cdot \cdot T_{n}=I.$$

The identity matrix is denoted by *I* in the formula above, and the relative transformation matrix between the observed data frames is denoted by $T_{i}$.

#### 3.2.3. Building a Dynamic Bayesian Network-Based Graph Model

**Figure 9:**
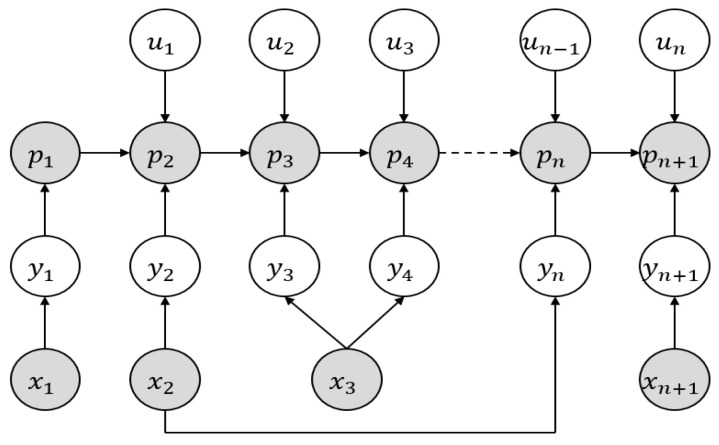
Dynamic Bayesian network representation-based graph structure.

Dynamic Bayesian networks are currently widely used in research domains like pattern recognition, computer vision, and signal processing. The time-varying random variables and their interrelation can be expressed simply by using dynamic Bayesian networks. Typically, the graph model represented by dynamic Bayesian networks consists of two kinds of nodes: implicit nodes that correspond to unobservable parameter variables and explicit nodes that match observable parameter variables. Figure 9 depicts the graph model structure for real-time reconstruction based on dynamic Bayesian network expression, with white nodes standing in for visible explicit parameter variables. As an illustration, consider the observation output information $y_{i}$ (*i =* 1, …, *n* + 1) of feature points and the control input information $u_{i}$ (*i =* 1, …, *n*) of mobile robots. Gray nodes stand in for the unobservable implicit parameter variables. Edges in the graph, such as the pose information $p_{i}$ (*i =* 1, …, *n* + 1) of mobile robots and the position information $x_{i}$ (*i =* 1, …, *n* + 1) of feature points, describe the conditional dependency relationship between nodes.

**Figure 10:**
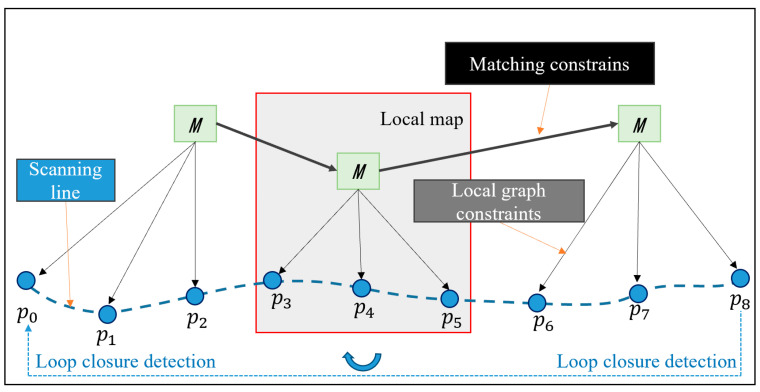
Structure of the Optimization Framework.

The moving robots current pose is represented by the constraint equation nodes that were built in order to optimize the structure in this paper. The point cloud matching result parameters and pose state information are represented by the edge. Using the nonlinear least squares approach to solve the optimization result that minimizes the error and creates an objective function of error through the establishment of a covariance matrix. Figure 10 displays the structure of the established graph optimization model. M stands for the local map among them, and $p_{0},p_{1},\ldots ,p_{n}$ for the LiDAR pose parameter data, respectively.

By incorporating node operators and edge error functions, parameter analysis is carried out by further utilizing the G2O framework to address local and global graph optimization issues. The purposeful function is:

(19) $$W=\sum_{\left(i,j\right)}{\left(D_{ij}-{\bar{D}}_{ij}\right)}^{T}C_{ij}^{-1}\left(D_{ij}-{\bar{D}}_{ij}\right).$$

$P_{i}=(x_{i},y_{i},z_{i},\theta_{xi},\theta_{yi},\theta_{zi})$ represents a set of points in the point cloud. $P_{j}=(x_{j},y_{j},z_{j},\theta_{xj},\theta_{yj},\theta_{zj})$ indicates neighboring nodes. $\left(x_{i},y_{i},z_{i}\right)$ indicates the point cloud’s coordinate value. ${(\theta }_{xi},\theta_{yi},\theta_{zi})$ indicates the rotation angle of each axis. $C_{ij}$ indicates the covariance matrix. ${\bar{D}}_{ij}$ indicates the edge of the observation constraint, and $D_{ij}$ indicates the edge that has to be estimated.

(20) $$D_{ij}=\left({\bar{H}}_{i}∆P_{i}-{\bar{H}}_{j}∆P_{j}\right),$$

(21) $${\bar{D}}_{ij}={\left(M^{T}M\right)}^{-1}M^{T}Z,$$

(22) $$C_{ij}=s^{2}{\left(M^{T}M\right)}^{-1}.$$

An unbiased estimate of the error variance is represented by $s^{2}$ in the formula above. H depicts the matrix of correlation. The mistake at the corresponding location is denoted by ${\bar{Z}}_{k}$, and the concatenated matrix made up of ${\bar{Z}}_{k}$ is represented by Z. The discrepancy between the point cloud’s actual position and its predicted optimal position is denoted by $∆P_{i},\;\;∆P_{j}$. The computed decomposition matrix is represented by ${\bar{H}}_{i}\;and\;{\bar{H}}_{j}$. The decomposition matrix $M_{k}$ is used to determine the covariance of the point cloud, and the concatenated matrix M is made up of $M_{k}$.

Further linearize $D_{ij}$ in the form of correlation matrix: D=HP. D and P are, respectively, composed of $D_{ij}$ and ${\bar{H}}_{i}∆P_{i}$ in series. The objective function is expressed as:

(23) $$W={\left(\bar{D}-HP\right)}^{T}C^{-1}\left(\bar{D}-HP\right).$$

The block array C in the formula above is made up of $C_{ij}$, and the block array $\bar{D}$ is made up of $D_{ij}$ connected in series.

Supposing $G=H^{T}C^{-1}H$, $B=H^{T}C^{-1}\bar{D}$ then the sub items $G_{ij},\;B_{i}$ of G and B can be expressed as:

(24) $$G_{ij}=\left\{\begin{array}{c} \sum_{j=0}^{n}C_{ij}^{-1},\;\;i=j\\ C_{ij}^{-1},\;\;i\neq j \end{array}\right.,$$

(25) $$B_{i}^{-1}=\sum_{j=0,i\neq j}^{n}C_{ij}^{-1}{\bar{D}}_{ij}.$$

Lastly, a system of equations solved in order to define the optimization problem as B=GP. The optimum posture information is as follows:

(26) $$P_{i}={\bar{P}}_{i}-{\bar{H}}_{i}^{-1}∆P_{i}.$$

The current pose is denoted by ${\bar{P}}_{i}$ in the formula above. The difference between the current and estimated poses is represented by $∆P_{i}$, and the optimized position is denoted by $P_{i\;}$.

While the local maps are being constructed, the algorithm calculates a fast point feature histogram for each frame of the point clouds. In this paper, loop closure detection is executed with this histogram to improve the efficiency of the algorithm. The new constraints gained from loop closure detection will be added into the graph as new edges, which can effectively reduce the cumulative error during the robot movement. As shown in Figure 10, the robot travels sequentially from P0 to P8 and detects a marked similarity with P0 at point P8. Therefore, the P0–P8 position and attitudes constraints are established, and this constraint is added into the graph as a new edge. Finally, the local map positions and attitudes are optimized according to the graph optimization framework, and the local maps are stitched together to form a global map.

## 4. Experimentation

### 4.1. Experimental Performance in KITTI Dataset

**Figure 11:**
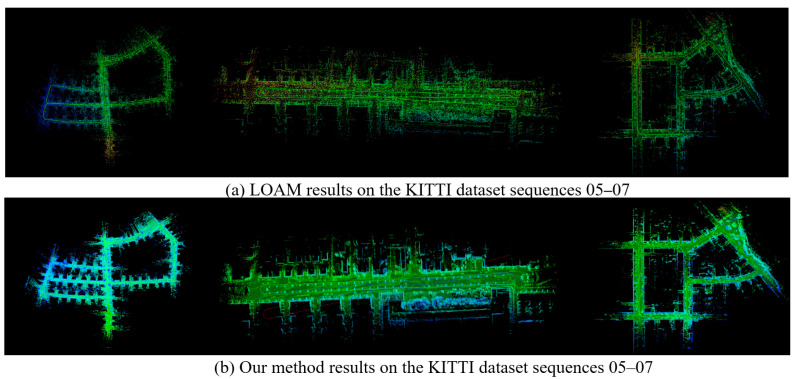
Experimental results on the KITTI dataset sequences 05–07.

This article validates the feasibility of our method on the 05–07 sequence of the KITTI dataset, and the results are shown in Figure 11. It can be seen that the 3D point cloud constructed by the algorithm proposed in this article has better performance. Especially at corners and loop nodes, the point clouds of each frame have better overlap.

**Figure 12:**
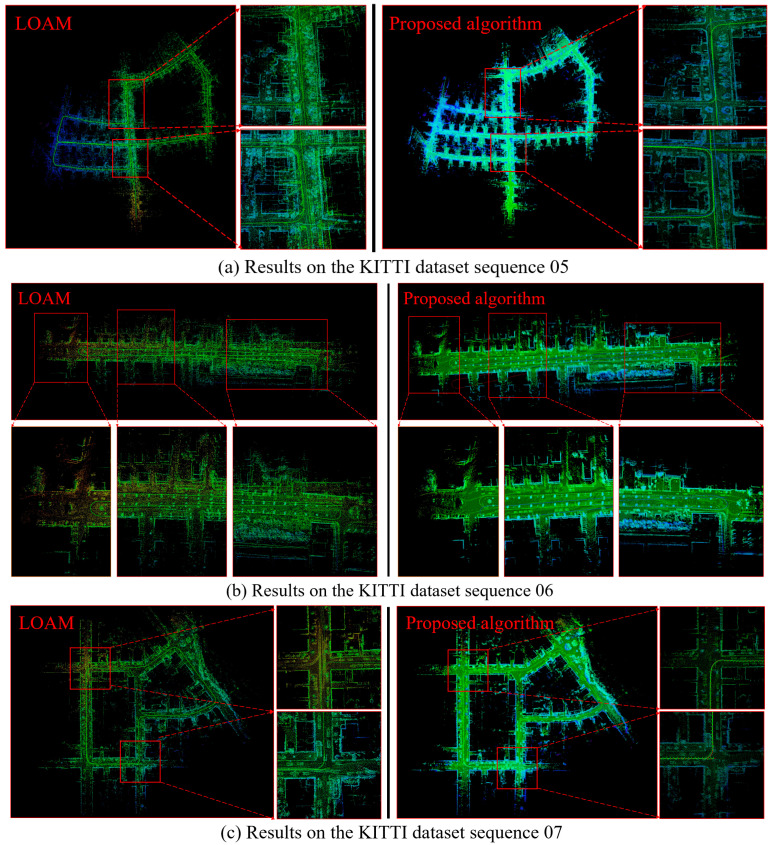
Enlarged details on the KITTI dataset sequences 05–07.

The specific details are enlarged, as shown in Figure 12. It can be seen that the algorithm proposed in this article has a better reconstruction effect, and the trajectory is more in line with the actual running state.

The comparison of APE (Absolute Percentage Error) and RPE (Relative Percentage Error) in the KITTI dataset experimental results is shown in Table 3.

### 4.2. Unstructured Terrain Environment Reconstruction Experimentation

**Figure 13:**
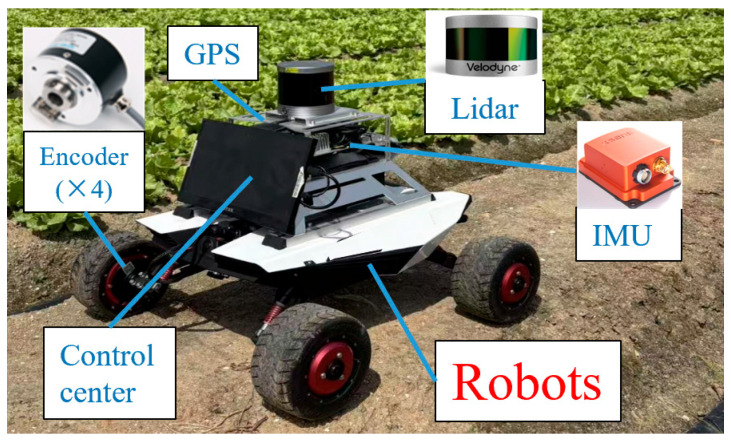
Experimental platform.

Figure 13 displays the experimental platform that was used to further validate the advantages of the algorithm proposed.

The primary sensors mounted on the robots are LiDAR, IMU, and Encoders. Table 4 displays the sensor’s precise parameters.

**Figure 14:**
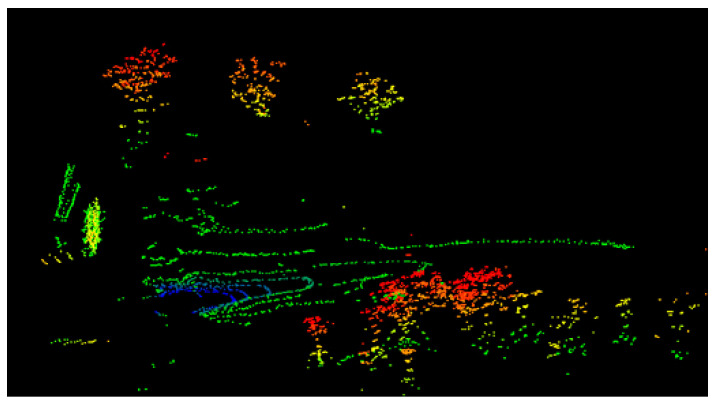
Construction effect of terrain point cloud.

The algorithm for processing LiDAR data was running on a portable computer with 32 GB of RAM, eight cores, and 2.3 GHz throughout the experiment. The mileage calculation method and the reconstruction algorithm operate on two different kernels simultaneously during the algorithm’s execution. Figure 14 displays the created local terrain point cloud. The reconstruction algorithm in this article has a calculation frequency of 1 Hz.

The aforementioned graphic illustrates how well the local terrain point cloud created by the technique suggested in this article can capture the features of the ground and trees in unstructured terrain conditions. It is challenging to assess the algorithm’s efficacy because of the limited quantity of point clouds in the created single frame local terrain point cloud. Longer reconstruction tests were therefore carried out in outdoor settings.

The LiDAR was mounted on a robot for the outdoor unstructured terrain environment experiment.

**Figure 15:**
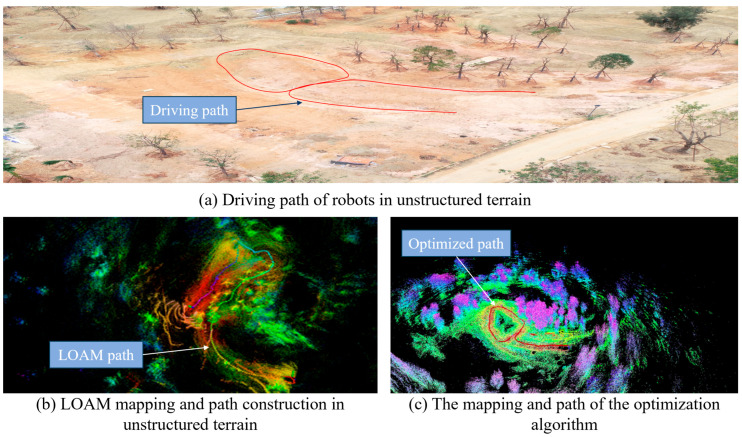
Comparison of reconstruction results in unstructured terrain.

Figure 15a depicts the robots driving path in an unstructured terrain environment; Figure 15b depicts the reconstruction effect and path of the standard reconstruction algorithm, LOAM, for a single LiDAR in the same environment. It is evident that the LOAM algorithm has exhibited considerable distortion and has not created a fully circular junction path because of the intricacy of the unstructured terrain environment and the ambiguous elements. Figure 15c illustrates the reconstruction impact and path of the optimization procedure suggested in this paper in an unstructured terrain environment. As can be observed, the reconstruction effect is more in line with the actual unstructured terrain environment, and the algorithm suggested in this article is more consistent with the driving trajectory of the robots.

Further ablation experiments of the proposed algorithm for module gain and runtime are conducted, and the results are shown in Table 5. The LOAM base model performed consistently over the course of the experiment. By adding the position and attitude optimization module alone, the accuracy is improved significantly. It can be observed that the unstructured environment has a greater influence on the robot position and attitude, which affects the accuracy of terrain modeling. With the addition of the graph optimization module alone, the improvement in accuracy is less significant, while the runtime is longer. The highest accuracy is achieved when both modules are added at the same time, although the runtime is consumed longer.

**Figure 16:**
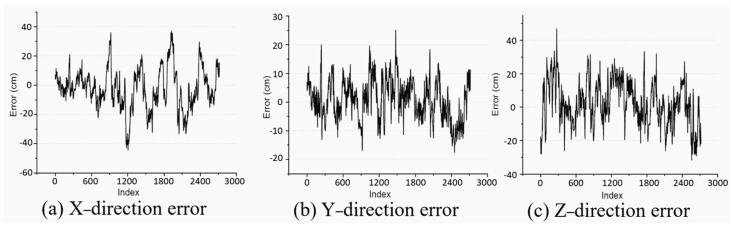
Analysis of position estimation error.

The specific error analysis on the unstructured terrain environment is shown in Figure 16. From the figure, it can be seen that the method proposed in this article has high accuracy in pose estimation. The average value of position estimation error is 9.16 cm.

## 5. Discussion

The experimental results in Section 4 demonstrate several important findings: Firstly, with the introduction of IMU information and the graph optimization algorithm, the algorithm proposed in this paper can achieve better 3D reconstruction performance. We believe that this performance improvement is not only attributed to the integration of IMU information, but also benefits from the fact that the graph optimization algorithm can optimize the one-to-one correspondence between point clouds. Secondly, we conducted comprehensive comparative experiments on the structured dataset, which effectively verified the effectiveness of the method we proposed. In addition, especially in experiments involving unstructured terrain environments, traditional algorithms have shown signs of failure, and the robots’ trajectory has significantly deviated from the true value. However, the algorithm proposed in this paper can not only effectively achieve the three-dimensional reconstruction of unstructured terrain point clouds but also maintain a high level of accuracy.

## 6. Conclusions

In order to calibrate the distortion of LiDAR and successfully mitigate the effects of nonlinear robots’ motion, this article first employs IMU to give robots motion information. Lastly, a thorough investigation of the reconstruction of the terrain in outdoor unstructured environment was carried out. The results of the experiments demonstrate that the algorithm presented in this research is able to reconstruct the unstructured terrain environment with more accuracy.

## Figures and Tables

**Table 1:** Parameters of feature point detection algorithm.

Curvature Threshold	Neighborhood Size	Number of Crucial Corners	Number of Crucial Plane Points	Breakpoint Distance Threshold	Breakpoint Radian Threshold	Parallel Point Threshold	Clustering Threshold
0.1	10	2	4	0.1	0.1	0.0002	0.2 m

**Table 2:** Parameters of the feature point alignment algorithm.

Threshold for the Number of Corner Points	Threshold for the Number of Plane Points	Number of Iterations	Local Point Cloud Size	Neighborhood Size
10	100	25	5 m × 5 m × 5 m	5

**Table 3:** The comparison of APE and RPE results.

	KITTI Dataset Sequence 05				KITTI Dataset Sequence 06				KITTI Dataset Sequence 07			
	LOAM		Our Method		LOAM		Our Method		LOAM		Our Method	
	APE	RPE	APE	RPE	APE	RPE	APE	RPE	APE	RPE	APE	RPE
Max	11.40	6.51	10.81	5.80	11.70	5.92	11.18	4.24	0.73	3.40	0.72	2.98
Min	1.12	0.01	1.08	0.01	0.68	1.79	0.55	0.62	0.12	0.01	0.08	0.01
Mean	2.85	1.34	2.80	1.26	4.61	2.30	4.63	2.07	0.40	0.96	0.39	0.93
Std	1.84	0.79	1.69	0.75	2.50	0.99	2.38	1.01	0.12	0.52	0.11	0.49

**Table 4:** Experimental platform sensor parameters.

Sensor	Model	Physical Quantities	Parameters
Lidar	Velodyne VLP-16 (Velodyne LiDAR, Inc., San Jose, CA, USA)	3D point cloud data	Measurement range: 0–100m Accuracy: ±3 cm Angular resolution (horizontal): 0.1–0.4° Angular resolution (vertical): 2.0°
IMU	Xsens MTi-700 (Xsens Technologies B.V., Enschede, The Netherlands)	Euler angle	Repeatability deviation: 0.1°/s Sampling frequency: 10 KHz
Encoder	E6B2-CWZ6C (Omron Automation and Safety, Kyoto, Japan)	Speed	Precision: 1000 P/R Maximum speed: 6000 r/min Maximum response frequency: 100 KHz

**Table 5:** Ablation performance of optimization algorithms.

LOAM (Baseline)	Position and Attitude Optimization Module	Graph Optimization Module	APE	RPE	Running Time/ms
√	-	-	35.38	44.61	26.79
√	√	-	13.56	12.88	28.63
√	-	√	27.39	19.62	46.89
√	√	√	6.55	5.29	63.52

## Data Availability

The data presented in this study are available on request from the corresponding author.
